# Confirming the normal range for Mebrofenin liver function indices

**DOI:** 10.22038/aojnmb.2025.90333.1666

**Published:** 2026

**Authors:** Kathy P Willowson, Geoff Schembri, Elizabeth A Bailey, Andrew Markewycz, Vivian Chan, Andrew Cluff, Heidi Fearnside, Timothy Kwong, Charlotte Yee, Andrew See, Dale L Bailey

**Affiliations:** 1 Department of Nuclear Medicine, Royal North Shore Hospital, Sydney, Australia; 2 Institute of Medical Physics, The University of Sydney, Sydney, Australia; 3 North Shore Radiology and Nuclear Medicine, North Shore Private Hospital, Sydney, Australia; 4 Department of Radiology, St Vincents Hospital, Sydney, Australia; 5 Faculty of Medicine and Health, University of Sydney, Sydney, Australia

**Keywords:** Mebrofenin, Liver, Hepatobiliary scintigraphy, Portal vein embolisation Radioembolisation

## Abstract

**Objective(s):**

To establish normal ranges for [^99m^Tc] Tc-Mebrofenin hepatobiliary scintigraphy (HBS) indices of liver function and the associated uncertainty in clinical measures due to region placement.

**Methods:**

47 patients referred for gallbladder assessment with no history of liver disease were included in the study. Patients underwent dynamic HBS following injection of 200 MBq of [^99m^Tc] Tc-Mebrofenin at 10 seconds/frame for 36 frames. Analysis was performed by 5 experienced technologists using in-house software (MIM Software, Cleveland, Ohio) to establish blood pool (BP) clearance rate (%/min), blood clearance half-time (min), mebrofenin liver uptake rate (MUR) (%/min) and MUR normalised to body-surface-area (MUR_BSA_) (%/min/m^2^). Limits of normal ranges (95%) were established and correlation of functional indices with age was investigated. Analysis was repeated after a minimum of 4 weeks to establish intra-user variability and uncertainty associated with measures.

**Results:**

Data were collected for 27 women and 20 men, with an age range of 20 – 81 years. Twenty-seven patients were aged over 50 and 20 patients were aged below 50. The data were found to have a normal distribution. The mean values derived for the entire cohort for BP clearance rate, blood clearance half time, MUR, and MUR_BSA_ were 16.8±2.6 %/min, 4.3±0.7 min, 14.4±2.0 %/min and 8.0±1.5 %/min/m^2^, respectively. No significant difference in values was found between age groups and no correlation between liver function and age was found. The lower range of normal for MUR_BSA_ was established as 5.1%/min/m^2^, with clinical measures expected to have an uncertainty of ±0. 6 %/min/m^2^.

**Conclusion:**

The MUR_BSA_ value for a patient with normal liver function can be expected to be approximately 8.0±1.5 %/min/m^2^, with a lower limit of normal function at 5.1 %/min/m^2^. Patients receiving liver surgery or treatment that express MUR_BSA_ values below this may be at higher risk and should potentially be treated with caution.

## Introduction

 [^99m^Tc] Tc-Mebrofenin is a radiopharma-ceutical that allows for the quantification of hepatic function. Clearance of [^99m^Tc] Tc-Mebrofenin as a reproducible measure of both global and regional liver function were first described by Ekman (1) with further development by others (2, 3). 

 Hepatobiliary scintigraphy (HBS) using [^99m^Tc] Tc-Mebrofenin is a unique liver function test as it allows for additional regional assessment as opposed to only a global measure of function that is derived from standard liver function tests (LFTs) such as biochemical assays, CT volumetry or indocyanine green (ICG) clearance rate. It was first introduced to aid in the provision of estimates of liver reserve following portal vein embolisation (PVE) prior to surgery to stratify risk of post-surgical morbidity (4). More recently it has also found a role in treatment planning for radio-embolisation (RE), particularly for selective treatments when assessing functional burden in spared liver parenchyma to guide tolerable absorbed radiation dose (5). 

 While the use of [^99m^Tc] Tc- Mebrofenin HBS is growing and methodology guidelines have recently been published (6), little is understood about normal ranges that can be expected in the healthy population when following such methodology, an important parameter to guide clinical management and treatment decisions in patients. In 2015, Tann et al (7) presented a study based on 129 cases (heavily skewed to the female population with 126 female subjects) to establish the Mebrofenin clearance rate in patients with no known chronic or acute liver or biliary disease. Results suggested a normal range of Mebrofenin clearance rate of 8.8±1.8 (SD) %/min (not normalised to patient BSA) with good inter-observer reliability noted. A 2016 study (8) reported a decline in liver function with age following a linear trend and the associated expected normal range as 8.5±2.1 %/min/m^2^, however the data were derived from patients diagnosed with hepatic metastases and benign tumours.

 This study aims to assess normal ranges for [^99m^Tc] Tc-Mebrofenin HBS in a population of patients being referred for gallbladder dynamic studies with no known liver disease following recommended acquisition and region analysis techniques (6). The study also seeks to establish the uncertainty in individual clinical measures based on intra-user variation in region placement.

## Methods

 All patients were adults undergoing assessment of gallbladder function with presumed normal liver function based on clinical history including no history of liver disease. No selection criteria were used and all patients undergoing gallbladder assessment in the study time frame were considered. Patients were administered 200 MBq of [^99m^Tc] Tc-Mebrofenin and dynamic data were acquired for 10 seconds/frame over 36 frames using a 128×128 matrix on a dual detector Siemens Symbia Evo (Siemens Healthineers, Erlangen, Germany). Geometric mean data were analysed using in house software (MIM Software, Cleveland, Ohio) according to recently published clinical practice guidelines (6). Blood pool (BP) clearance rate (%/minute), blood clearance half time (minutes), Mebrofenin liver uptake rate (MUR) (%/minute) as first described by Ekman (1), and MUR normalised to body surface area (MUR_BSA_) (%/minute/m^2^) were derived. 

 All studies were independently analysed by five experienced technologists on two separate occasions, spaced more than 4 weeks apart. Comparison of inter-user results allowed for the derivation of normal indices including expected uncertainties, whilst comparison of intra-user results was used to indicate uncertainty in a single clinical measurement. Each of the above parameters were derived for the total patient cohort as well as patients grouped into age brackets 20–50 years and >50 years to investigate an age dependency, which was done via linear regression. The difference in indices derived between each age cohort was also investigated for significance via an independent T-test (2025 GraphPad Software). The limits of the normal range (95%) were also established. 

## Results

**Figure 1:**
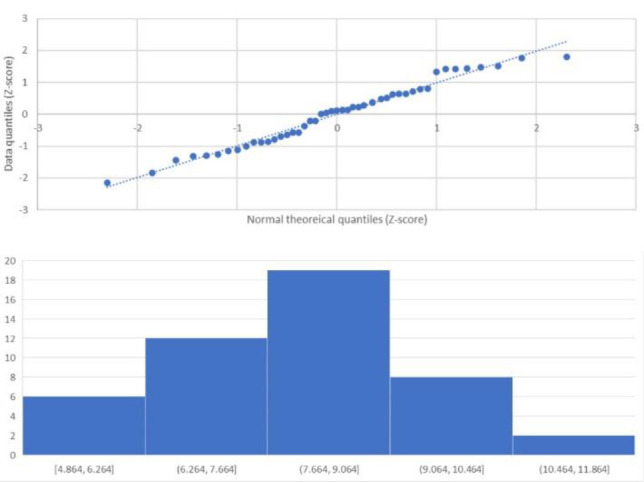
Analysing the full cohort of MURBSA derived data for a normal distribution, represented by a linear fit on the QQ plot (**A**) and an approximately bell-shaped histogram (**B**)

 The study cohort was comprised of 27 women and 20 men, with an age range of 20-81 years. Twenty-seven patients were aged over 50 years and 20 patients were aged below 50 years. The data was found to have a normal distribution based on a histogram and QQ plot (Figure 1) suggesting it is a representative cohort for analysis. 

 The derived indices for hepatic function and normal ranges (95%) are shown in Table 1 and 2, respectively, with an average MUR_BSA_ value of 8.0±1.5 %/min/m^2^ for the entire cohort.

**Figure 2:**
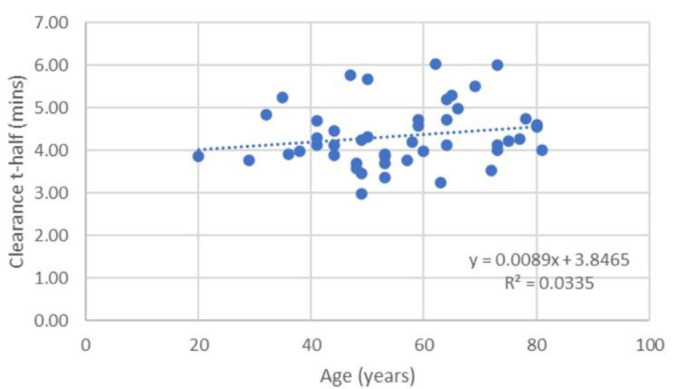
The relationship between clearance t-half and subject age. The fitted linear regression suggests no significant decline in function is demonstrated with age

 No relationship was found between age and declining liver function, as demonstrated by the lack of correlation seen in the linear regression fit to data in Figure 2. Similarly, no significant difference was demonstrated when comparing indices between the under 50 years cohort and the over 50 years cohort (p=0.46 for MUR_BSA_).

 The intra-user variability derived from repeat analysis by all five experienced technologists suggests an uncertainty associated with measures of BP clearance rate, blood clearance half time, MUR and MUR_BSA_ equal to 1.4 %/min, 0.44 mins, 0.94 %/min and 0. 6 %/min/m^2^, respectively.

**Table 1:** Derived hepatic function indices with associated uncertainty derived from standard deviation across measures

	**Mean Blood Pool Clearance Rate (%/min)**	**Mean Blood Clearance ** **half-time (mins)**	**Mean Liver MUR (%/min)**	**Mean Liver MURBSA (%/min/m2)**
**Total Cohort**	16.8 ± 2.6	4.3 ± 0.7	14.4 ± 2.0	8.0 ± 1.5
**20 – 50 yrs**	17.2 ± 2.5	4.3 ± 0.7	14.8 ± 2.0	8.2 ± 1.4
**> 50 yrs**	16.4 ± 2.6	4.4 ± 0.7	14.1 ± 1.9	7.9 ± 1.5

**Table 2:** Recommended normal limits based on derived normal ranges (95%) for liver function indices

	**Lower Limit of ** **Blood Pool Clearance Rate (%/min)**	**Upper Limit of Blood Clearance half-time (mins)**	**Lower Limit of Liver MUR (%/min)**	**Lower Limit of Liver MURBSA (%/min/m2)**
**Total Cohort**	11.8	5.8	10.5	5.1
**20 – 50 yrs**	12.3	5.6	10.9	5.4
**> 50 yrs**	11.4	5.8	10.3	4.9

## Discussion

 The data agree reasonably well with published values based on a cohort with metastatic liver disease (8), which are stated as 8.5±2.1 %/min/m^2^, however in contrast to this publication, no significant decline in liver function measures with age was observed. This may be due to the limitations of a small cohort, or perhaps due to differences in the normal population being studied, in this case patients with no history of liver disease compared with a study based on patients with known liver metastases. The data are also lower than Tann’s (7) published results of 8.8±1.8 %/min, however these data have not been subject to normalisation to patient BSA. Given both studies were performed prior to the broader publication of accepted methodology guidelines, minor differences in data acquisition and analysis may also be a contributing factor.

 No significant difference was seen in liver function for patients over the age of 50 when compared to those below the age of 50 as been suggested by Cieslak et al (8), however a larger cohort may be needed to demonstrate this. 

Repeat analysis demonstrating intra-user variability due to region placement suggests an uncertainty associated with MUR_BSA_ of 0. 6 %/min/m^2^, indicating a robust measurement technique followed by technologists. Such uncertainty can be applied to individual clinical measures of liver function indices when comparing patient specific values to the derived normal ranges to assess whether or not a given patient falls within acceptable limits of liver function.

 The limits of normal function can be compared to individual patient results and are expected to act as a guide when making clinical decisions regarding patient management. Such values are particularly useful when interpreting FRL function prior to surgery following PVE, and to add confidence to RE dose prescription for selective treatments. 

## Conclusion

 The normal range of [^99m^Tc] Tc-Mebrofenin HBS indices have been established. Such values can guide clinical management for surgery and RE patients. Patients with a MUR_BSA_ below 5.1 %/min/m^2^ are considered potentially at risk and should be treated with caution in combination with clinical interpretation of alternative liver function tests.
